# Soft metamaterial with programmable ferromagnetism

**DOI:** 10.1038/s41378-022-00463-2

**Published:** 2022-12-06

**Authors:** Kerem Kaya, Emre Iseri, Wouter van der Wijngaart

**Affiliations:** Division of Micro and Nanosystems, KTH Royal Institute of Technology, Stockholm, 100 44 Sweden

**Keywords:** Materials science, Engineering

## Abstract

Magnetopolymers are of interest in smart material applications; however, changing their magnetic properties post synthesis is complicated. In this study, we introduce easily programmable polymer magnetic composites comprising 2D lattices of droplets of solid-liquid phase change material, with each droplet containing a single magnetic dipole particle. These composites are ferromagnetic with a Curie temperature defined by the rotational freedom of the particles above the droplet melting point. We demonstrate magnetopolymers combining high remanence characteristics with Curie temperatures below the composite degradation temperature. We easily reprogram the material between four states: (1) a superparamagnetic state above the melting point which, in the absence of an external magnetic field, spontaneously collapses to; (2) an artificial spin ice state, which after cooling forms either; (3) a spin glass state with low bulk remanence, or; (4) a ferromagnetic state with high bulk remanence when cooled in the presence of an external magnetic field. We observe the spontaneous emergence of 2D magnetic vortices in the spin ice and elucidate the correlation of these vortex structures with the external bulk remanence. We also demonstrate the easy programming of magnetically latching structures.

## Graphical abstract

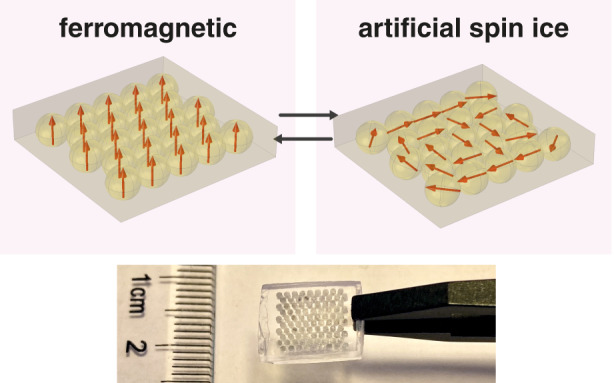

## Introduction

Magnetic composite materials have been investigated for various applications, including data storage^1^, robotics^2–6^, and biomedicine^4^. To create soft matter composites with magnetic properties, microscale^2,4,7^ or nanoscale^1,8^ magnetic particles are embedded in a polymer matrix. For small enough (thermal) particles, Néel relaxation generates superparamagnetic properties^9^. Magnetopolymers with large remanence are typically formed with large (athermal) hard-magnetic particles; the particle orientation is controlled with an external magnetic field during polymerisation^10^ and mechanically fixated after material synthesis^11^. Because their Curie temperature exceeds the thermal degradation temperature of their polymer matrix, the remagnetisation of such ferromagnetic magnetopolymers requires degaussing. Thus, the functionalities of these magnetopolymers are limited, and they are only permanently programmed during manufacturing.

Song et al.^12^, Lin et al.^13^ and our team^14^ independently developed new types of magnetopolymers that embed athermal ferromagnetic particles in droplets of low melting point materials in polymer matrices. Above the melting point *T*_*m*_, the particles have rotational freedom. The uniqueness of these composites exists in their easily reprogrammable magnetisation profiles. This behaviour follows from the fact that particles (1) are athermal, (2) have Curie temperatures above the droplet melting point, and (3) are fixated in solid droplets while possessing full rotational freedom in molten droplets. This easy reprogramming is a critical characteristic for such materials to be used in a wide range of applications^15^, including reconfigurable soft grippers^7,16^, locomotion robots^17–19^, dynamic sensors, and actuators^20,21^; this characteristic allows scaled production and reconfiguration on demand. Thin and flexible magnetic polymer materials have further been demonstrated with origami-based folding sequences^22,23^ to program nonuniform magnetisation patterns on the sample. Flexible electronic circuits with different functions, such as flexible antennas, energy harvesting devices^23^, soft centrifugal pumps^24^ and soft vibration sensors^25^, have been shown with origami-enabled soft magnetic materials.

We describe the magnetic behaviour of soft materials with programmable ferromagnetism, demonstrate the easy magnetisation and demagnetisation of these magnetopolymers, and study the coupling between the material microstructure and the macroscale magnetic properties.

## Results and discussion

**Fig. 1:**
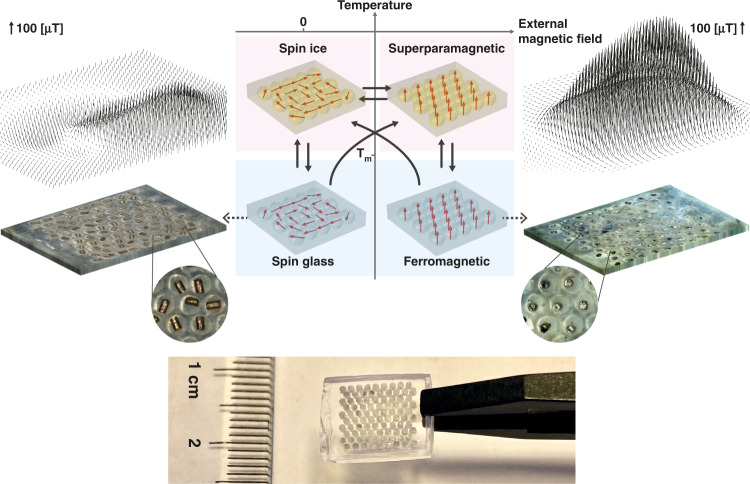
Soft metamaterial with programmable ferromagnetism. Middle bottom: photograph of a polymer magnetic metamaterial sample. Left and right: photographs of the top surface of a metamaterial sample in a low-remanence spin glass state with in-plane particle magnetisation (left bottom) and in a high-remanence ferromagnetic state with out-of-plane particle magnetisation (right bottom). The vector plots show the magnetic field, $\vec B$, measured one lattice unit distance, *c*, above the material centre planes of these samples. Middle top: Schematic of the four material states at different temperatures and external magnetic fields, with solid black arrows indicating the possible phase transitions

We performed experimental and numerical simulations on solid polymer matrices containing arrays of droplets of low melting point phase change materials (PCMs) that each trap one magnetised ferromagnetic particle (Fig. 1). The droplets were ordered in a 2D triangular lattice according to the highest droplet packing density. The PCM’s melting point determines the material’s blocking temperature: below the melting point, the particles are mechanically fixated (blocked); above the melting point, the particles rotate freely and align their magnetisation to an external field. Thus, unlike superparamagnetic nanoparticle composites, rotation-induced ferromagnetism decouples the Néel relaxation time and blocking temperature. Above the melting point, the full rotational freedom results in a superparamagnetic state. Heating in the presence of a magnetic field aligns the magnetisation of the particles with the field; after cooling, this process results in a ferromagnetic state characterised by a significant external remnant field. By heating in the absence of a magnetic field, the material evolves to a lower magnetic energy state through the magnetic particles rotating such that their magnetisation is in the material plane, thus forming a 2D dipole XY artificial spin ice. In contrast to spin ices in an Ising model with binary spin states, “spin” in our material refers to the magnetisation vector of every particle having full in-plane rotational freedom. After cooling below the blocking temperature, the spin ice is blocked and forms a spin glass state characterised by a low external remnant field. The material is controllably and repeatedly “reprogrammed” between stable ferromagnetic, superparamagnetic, spin ice and spin glass states.

Compared to multiparticle-per-droplet materials^12,13^, our single particle-per-droplet metamaterial uniquely does not depend on the intradroplet particle arrangements. For a given fraction of hard magnetic materials per droplet, the net magnetisation of multiparticle droplets reaches that of single-particle droplets only if all intradroplet particle magnetisations point in the same direction, which is a nontrivial state. Therefore, we speculate that our single-particle materials can feature larger bulk magnetisation than multiparticle materials.

**Fig. 2:**
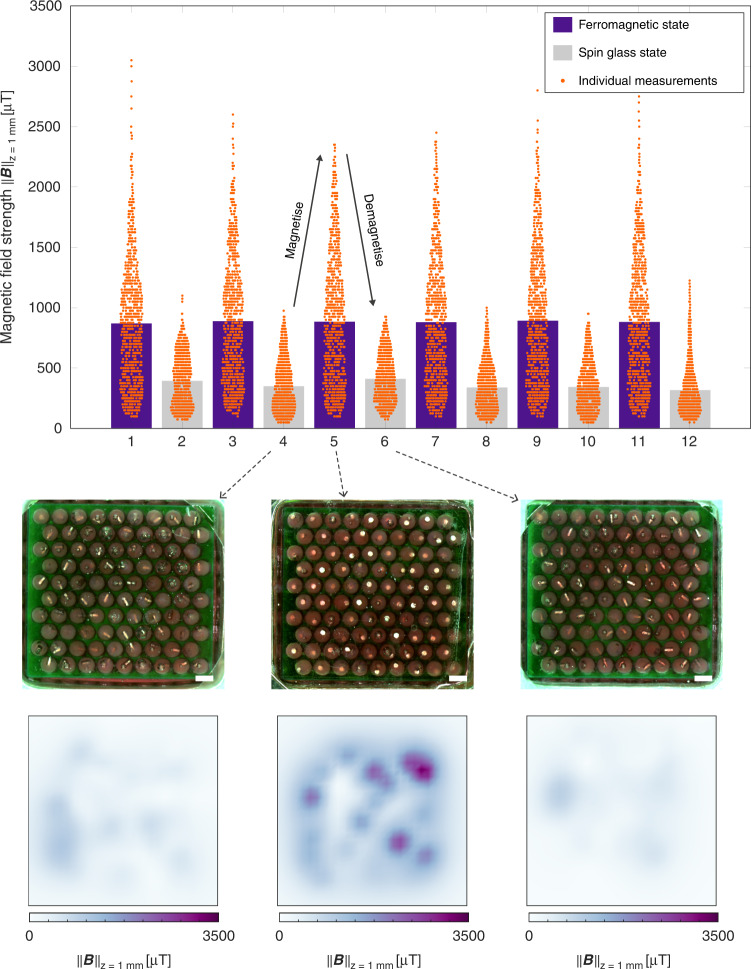
Magnetising and demagnetising. The bar plot shows the measured area-averaged bulk magnetic field strength, $\left| {\left| {\vec B} \right|} \right|_{z = 1mm}$, of a material sample at one lattice unit distance, *c*, above the material centre plane after repeated magnetising (purple) and demagnetising (grey) cycles. The scatter plots (orange) show individual magnetic field strength measurement values. The insets show top view photographs of the spin ice and superparamagnetic states before freezing (middle, scale bars are 1 mm) and the measured magnetic field strength $\left| {\left| {\vec B} \right|} \right|_{z = 1mm}$for the equivalent spin glass and ferromagnetic states (bottom)

We fabricated a demonstrator material by moulding a layer of thiol-ene polymer to contain a 9 × 10 array of hollow cylindrical cells (height 1 mm, diameter 0.75 mm) in a triangular lattice with a lattice unit distance *c* = 1 mm; filling each cell with a phase change material with the melting point *T*_*m*_ = 42 *°*C (RT42 phase change material, Rubitherm, Germany); and placing a single cylindrical magnetic N52 neodymium particle (height 500 µm, diameter 300 µm) in every cell. In the presence of an external out-of-plane magnetic field (placing the demonstrator on top of a permanent 76.6 mT neodymium magnet), the subsequent heating and cooling magnetises the prototype by aligning all particles in the out-of-plane *z*-direction. Heating and cooling outside an external magnetic field result in the magnetic particles rotating into the plane of the material to form a spin glass state, thus demagnetising the material (Supplementary Movies S1 and S2). We measured the magnetic field distribution by placing material samples below a robotic *x*–*y*–*z* stage and scanning the space above the material using an LSM9DS1 inertial measurement unit (IMU) sensor (STMicroelectronics, Switzerland). We found the average magnetic field strength at the demonstrator surface (distance *c* above the material centre plane) to be 0.879 ± 0.009 mT (*n* = 6) for the ferromagnetic state and 0.355 ± 0.036 mT (*n* = 6) for the spin glass state (Fig. 2).

We performed finite element simulations of arrays of spherical ferromagnetic particles with magnetic particle radii *r* and lattice unit distances *c* (Supplementary Fig. S1). Where not specified otherwise, simulations were performed with spherical particle radius *r* = *c/2* and arbitrary values of the magnetic particle remanent flux density *M*_*p*_ = 10*T* and relative permeability of 200.000. We started by aligning the magnetisation of all the particles in the *z*-direction. We then computed the magnetic field distribution and let the particles rotate one step in the direction of the magnetic momentum exerted on them. We repeated this computation and rotation until the magnetic energy of the system did not further decline (Supplementary Movie S3). As in the demonstrator experiments, the particle magnetisation directions evolved towards an in-plane spin ice state. The average remnant magnetic field at a distance *c* above the simulated material is 3.9 ± 0.3 (*n* = 12) times higher for the ferromagnetic state than for the spin glass state.

We further performed a simulation with parameters fitted close to those of the experiment, i.e., with spherical radius parameter of *r* = 0.2036 mm (resulting in the same volume fraction of magnetic material in the metamaterial), a magnetic particle remanent flux density of *M*_*p*_ = 1.45 T, and a relative permeability of 1.05 (Supplementary Fig. S2). We found an area-averaged external magnetic field $< \vec B_{sg} > _{{{{\mathrm{z}}}} = {{{\mathrm{c}}}}}$ 6.2 times larger than the experimental results, where we notate <*F*>_Π_ for the area-averaged magnitude of a field *F* in a plane Π ($< F > _{\Pi} \equiv \frac{1}{A}{\oint} {F \cdot dA}$ with *A* the area of Π). We speculate that the differences in the magnetic field strength values between the experiment and simulation stem from the different particle shapes (spherical vs. cylindrical) and from small in-plane shifts of the magnetic particle centres relative to the centre of their wells in the experiments (simulated particles are fixated at the centre position of their well).

**Fig. 3:**
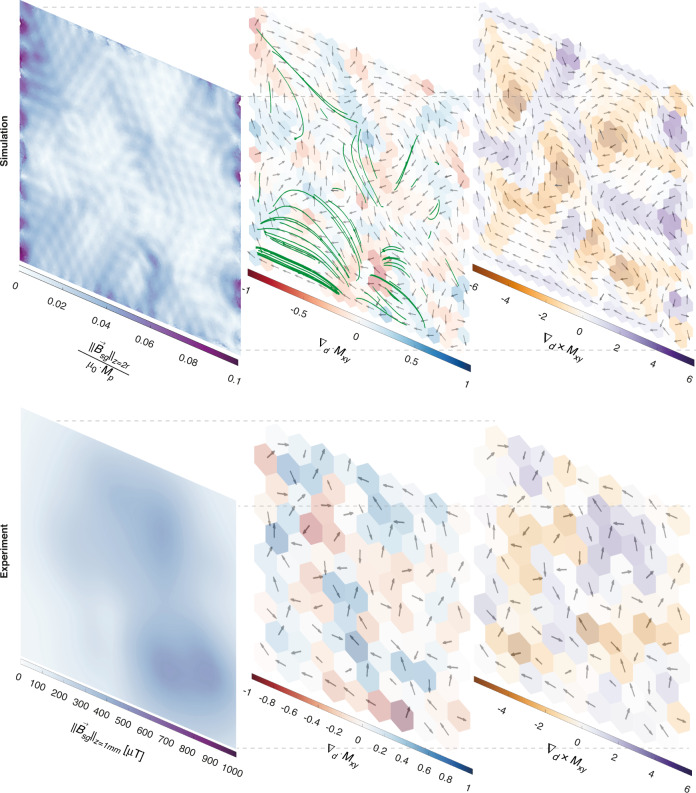
Simulated (top) and measured (bottom) in-plane magnetisation and remnant magnetic fields of a spin glass. Left: surface plot of the magnetic field strength at a lattice unit distance *z* = *c* above the material centre plane (white‒purple colour scale). Middle: the in-plane magnetisation vector field, *M*_*xy*_ (black arrows), and its divergence, $\nabla _d \cdot M_{xy}$ (red‒blue colour scale). The green lines indicate the magnetic field lines where the magnetic flux is the highest in the region *z* > *c* above the material plane. The magnetic field lines couple through the material plane in regions of high or low divergence. Right: the in-plane magnetisation vector field, *M*_*xy*_ (black arrows), and its curl, $\nabla _d \times M_{xy}$ (brown‒purple colour scale). High curl values indicate the centre of magnetic vortices

For every experiment and simulation, the resulting frustrated magnetic particle arrangements in the spin glass state are unique (Supplementary Fig. S3). Different simulation outcomes from identical start conditions result from tiny differences in the software-generated mesh structure. The chaotic nature of the magnetic particle rotation amplifies tiny differences between the starting conditions into unique spin ice states. Because the particle magnetisations of the spin ice are in the material plane, the centre plane of the material is a mirror symmetry plane for the magnetic field above and below the spin ice. In both experiments and simulations, we observed the formation of in-plane magnetic vortices in the artificial spin ice (Fig. 3). For the simulations, we found that large rotation steps between simulations resulted in large vortices.

This intriguing spontaneous emergence of magnetic order lead us to investigate the relationship between the discrete 2D vector field of in-plane particle magnetisation, *M*_*xy*_, and the external magnetic field of the spin glass state, $\vec B_{sg}$. The large magnetic vortices obtained via simulations aided us in elucidating the magnetic behaviour. We divided the material xy-plane into tiled hexagon cells centred around each droplet. We defined discrete divergence $\nabla _d \cdot$ and curl $\nabla _d \times$ operators on normalized *M*_*xy*_ and distance vectors so that $\nabla _d \cdot {{{\boldsymbol{M}}}}_{xy} \equiv \frac{{\mathop {\sum }\nolimits_{j = 1}^6 \overrightarrow {{{{\boldsymbol{M}}}}_j} \cdot \overrightarrow {{{{\boldsymbol{d}}}}_j} }}{{M_p \cdot c}} = \mathop {\sum}\nolimits_{j = 1}^6 {\frac{{\Vert{\overrightarrow {{{{\boldsymbol{M}}}}_j}}\Vert .\Vert\overrightarrow {{{{\boldsymbol{d}}}}_j}\Vert .\cos \theta _j}}{{M_p \cdot c}}}$ and$$\nabla _d \times {{{\boldsymbol{M}}}}_{xy} \equiv \frac{{\left[ {\mathop {\sum }\nolimits_{j = 1}^6 \overrightarrow {{{{\boldsymbol{M}}}}_j} \times \overrightarrow {{{{\boldsymbol{d}}}}_j} } \right]}}{{M_p \cdot c}}.\overrightarrow {{{{\boldsymbol{i}}}}_z} = \mathop {\sum}\nolimits_{j = 1}^6 {\frac{{\Vert{\overrightarrow {{{{\boldsymbol{M}}}}_j}}\Vert .\Vert\overrightarrow {{{{\boldsymbol{d}}}}_j}\Vert .\sin \theta _j}}{{\begin{array}{*{20}{c}} {M_p} \end{array} \cdot c}}}$$, where $\cdot$ is the scalar vector product, *×* is the cross product, $\overrightarrow {{{{\boldsymbol{i}}}}_z}$ is the unit vector in the *z*-direction, $\overrightarrow {{{{\boldsymbol{d}}}}_z}$ is the displacement between the centre of the hexagon and the centre of the particle in a neighbouring cell, ***M***_*xy*_ is the magnetisation of the particle in the neighbouring cell, ***M***_*p*_ is the magnetisation magnitude of the particle, and *c* is the lattice unit distance, which we sum over the six adjoining cells. Cells with high curl (>2.5) are centres of magnetic vortices. Cells with medium curl (in the range 0.5 and 2.5) are boundaries between vortices with the same direction of rotation.

**Fig. 4:**
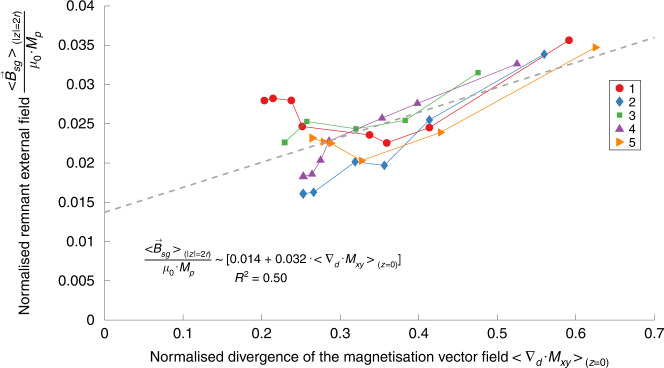
Correlation between the in-plane magnetisation field divergence and the bulk magnetic field strength for spin glasses. The dots indicate the normalised area-averaged remnant field strength, $\frac{{ < \vec B_{sg} > _{\left( {z = c} \right)}}}{{\mu _0 \cdot M_p}}$, versus the area-averaged magnitude of the magnetisation field divergence $< \nabla _d \cdot M_{xy} > _{(z = 0)}$ for the spin glass state of five simulation sequences (different marker shapes and colours) at different simulation steps. The magnetic field and magnetisation divergence tend to decrease with increasing simulation steps. The dotted line shows the linear regression of all values. The solid lines connect simulation steps within the same simulation sequence and are for visual guidance only

We can understand that magnetic vortices are regions where magnetic particles form low-energy closure domains. Where vortex regions meet, geometric constraints demand that the in-plane field diverges. For simulations with large vortex structures, we observe that the in-plane divergence is largest in regions where vortices with the same direction of rotation meet (Fig. 3 and Supplementary Fig, 2). Because the divergence of a 3D magnetic field is zero, regions of high in-plane divergence form magnetic poles for the out-of-plane field. This finding is confirmed by the external magnetic field lines originating from the high-divergence regions and by regions with large in-plane divergence coinciding with regions of high magnetic field strength at the material surface. Given the high average magnetic permeability inside the material plane, in-plane closure domain (magnetic vortex) patterns are energetically favoured over configurations where the magnetic field couples out-of-plane. We observe a trend towards fewer and larger vortices and decreased area-averaged divergence characteristics of the in-plane magnetisation for subsequent simulation steps (Fig. 4). The average magnetic field strength in a parallel plane at a distance *z* = *c* above or below the spin glass state correlates linearly with the divergence of the in-plane magnetisation:

$$\frac{{ < \vec B_{sg} > _{\left( {z = c} \right)}}}{{\mu _0 \cdot M_p}}\sim \alpha + \beta \cdot < \nabla _d \cdot M_{xy} > _{(z = 0)}$$

where $< \nabla _d \cdot M_{xy} > _{(z = 0)}$ is the area-averaged divergence of the in-plane magnetisation, *M*_*p*_ is the magnitude of magnetisation of a single particle, and *α* and *β* are fitting parameters that depend on the particle geometry, magnetisation, magnetic permeability, and distance to the material centre plane.

Because the material is a two-dimensional array of magnets, the material thickness decreases by decreasing the lattice unit distance, *c*. As a result, the total magnetic effect of the material scales linearly with the lattice unit size. Néel relaxation sets a physical limit to downscaling at magnetic particles of size a few tens of nanometres, below which the emergence of a stable ferromagnetic state is prohibited.

**Fig. 5:**
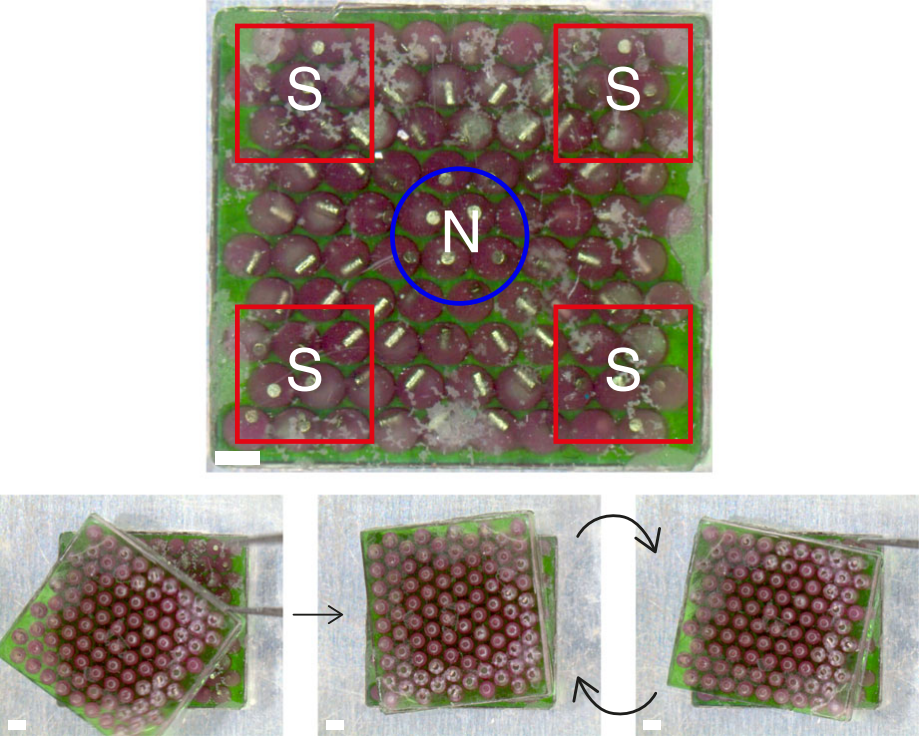
Top view photographs of the local magnetic programming (top) and the latching (bottom) of the magnetic material. Placing the materials on top of a fixture containing permanent magnets that are poled in opposite directions (the red squares and the blue circle indicate the positions of the magnets underneath the material) rearranges the magnetic particles in the material in a distinct pattern (top). Placing two similarly programmed magnetic materials on top of one another results in their spontaneous alignment and latching at 90-degree rotations (bottom). The scale bars are 1 mm

To demonstrate the local tuneability and potential niche applications, we readily programmed two material instances into latching magnets (Fig. 5). We stacked the materials in a nonuniform permanent magnetic field and briefly heated them above the droplet melting point to align their magnetic particles in matching patterns. (Supplementary Movie S4). After cooling, the two structures exhibited latching at 90-degree angles. (Supplementary Movie S5).

## Conclusion

In this work, we introduce and investigate ferromagnetic metamaterials with spatially distributed droplets of low melting point substances containing a single dipole particle. By varying the temperature across the melting point and controlling the external magnetic field, we reorient the particles to reprogram the material’s magnetic properties. Thus, the materials portray ferromagnetic behaviours with Curie temperatures defined by the droplet melting point, enabling magnetopolymers that uniquely combine high remanence and Curie temperatures below the polymer degradation temperatures. These important characteristics make them attractive for potential applications in robotics as smart, versatile and lightweight actuators. In addition, we evaluate the ferromagnetic behaviours of this class of materials, which may aid future magnetic actuator design. Future work can further investigate integrating reprogrammable magnetic metamaterials with specific lattices into multifunctional electronics/materials, including stretchable or curvilinearly shaped materials and their applications.

## Supplementary information

Supplementary Material for Soft Metamaterial with Programmable Ferromagnetism

Supplementary movie S1

Supplementary movie S2

Supplementary movie S3

Supplementary movie S4

Supplementary movie S5
