# Supplementary material for: Soft metamaterial with programmable ferromagnetism
Source: Microsyst Nanoeng. 2022 Dec 6;8:127. doi: 10.1038/s41378-022-00463-2 (PMC9722694; doi:10.1038/s41378-022-00463-2)
Supplement: Supplementary file 1 — Supplementary Material for Soft Metamaterial with Programmable Ferromagnetism [file 41378_2022_463_MOESM1_ESM.docx]

**Supplementary material: Soft metamaterial with programmable ferromagnetism**

Kerem Kaya^1^, Emre Iseri^1^ and Wouter van der Wijngaart^1*^

*^1*^Division of Micro and Nanosystems, KTH Royal Institute of Technology, Stockholm, 100 44, Sweden.*

*Corresponding author(s). E-mail(s): [wouter@kth.se](mailto:wouter@kth.se);

Contributing authors: [keremk@kth.se](mailto:keremk@kth.se); [iseri@kth.se](mailto:iseri@kth.se);

Supplementary movie S1 shows the "magnetisation" of a material sample by heating the sample after placing it on top of a permanent 76.6 mT neodymium magnet such that all particles align their magnetisation in the out-of-plane z-direction.

Supplementary movie S2 shows "demagnetisation" of a material sample by heating a magnetised sample in the absence of an external magnetic field such that all particles rotate their magnetisation from the out-of-plane z-direction into the xy-plane of the material where they form an artificial spin ice.

Figure S1 shows the triangular lattice structure of the magnets and their rotation with respect to their center points.


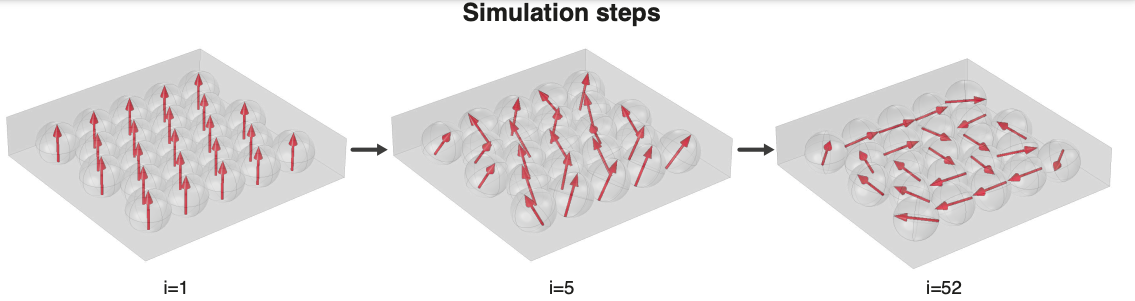


**Figure S1:** Overview of simulation setup steps and rotation of magnets. A triangular lattice of magnets is generated, and they are allowed to rotate freely from center points. Left: Initial state with iteration number 1, middle: iteration number 5, Right: Close to the end state, iteration number 52.

Supplementary movie S3 shows a top view of the magnetization vectors of the particles for subsequent simulation steps. The magnetisation vectors start from an out-of-plane z-direction and stepwise collapse into an in-plane artificial spin-ice configuration with magnetic vortices.


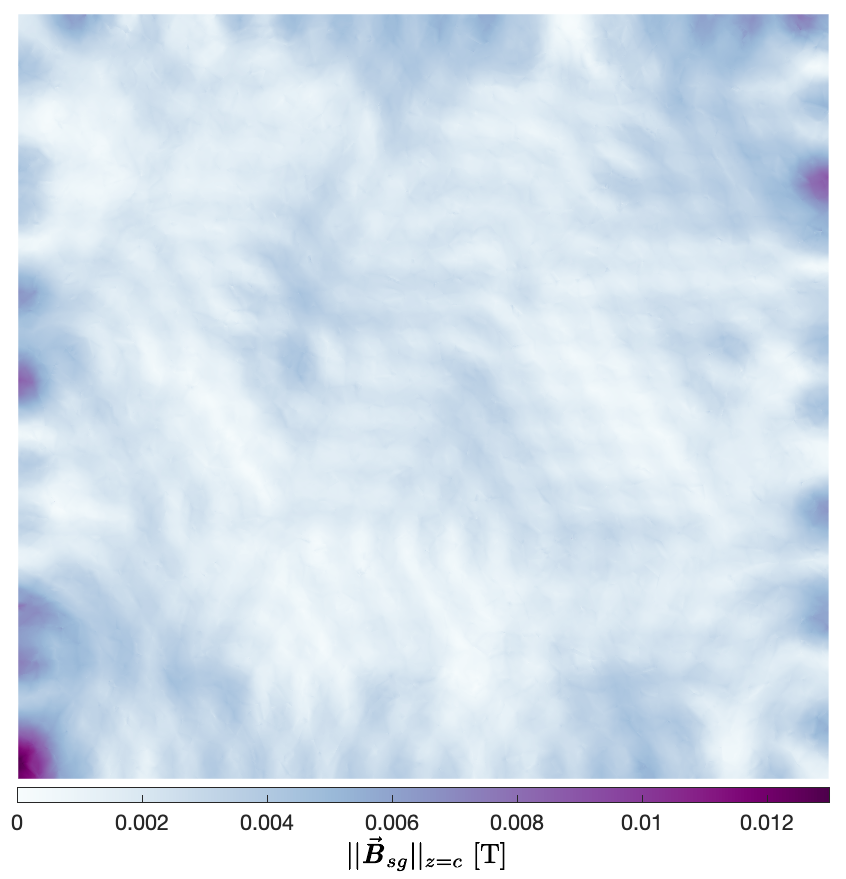


**Figure S2:** The simulation with the spherical parameter of radius $r=0.2036 mm$ (the same volume fraction of magnetic material in the metamaterial), magnetic particle remanent flux density, and relative permeability of 1.05 We found an area averaged remnant magnetic field strength, of 2.2 mT, 6.2 times larger than the experimental results.


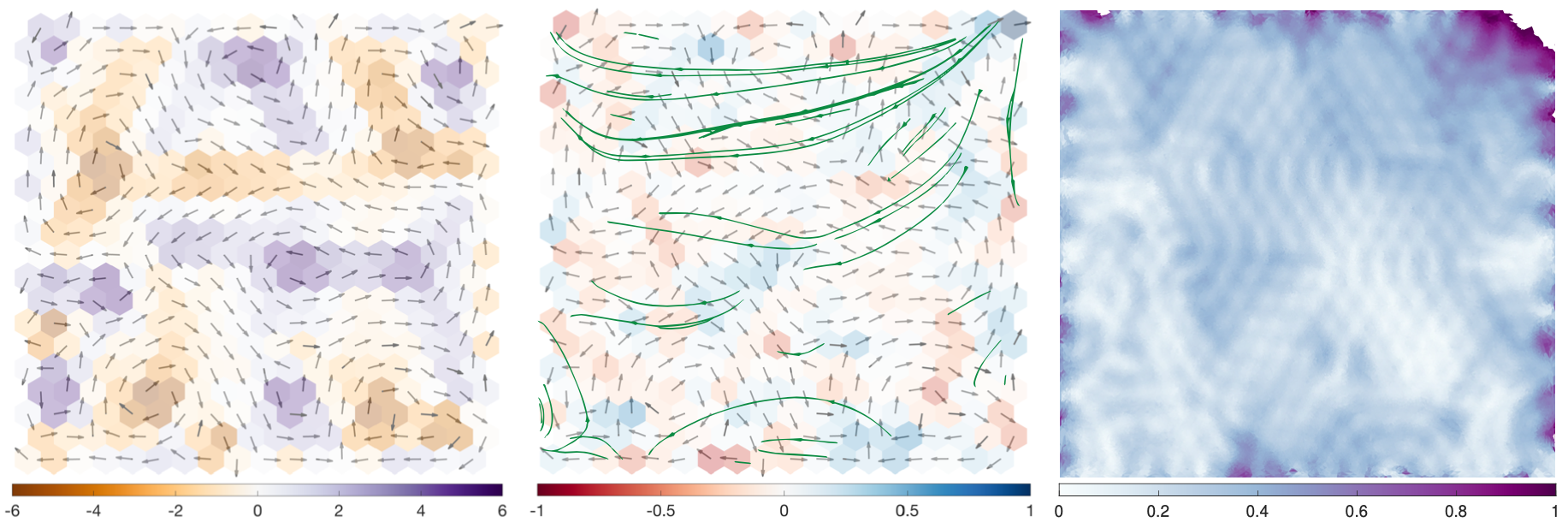


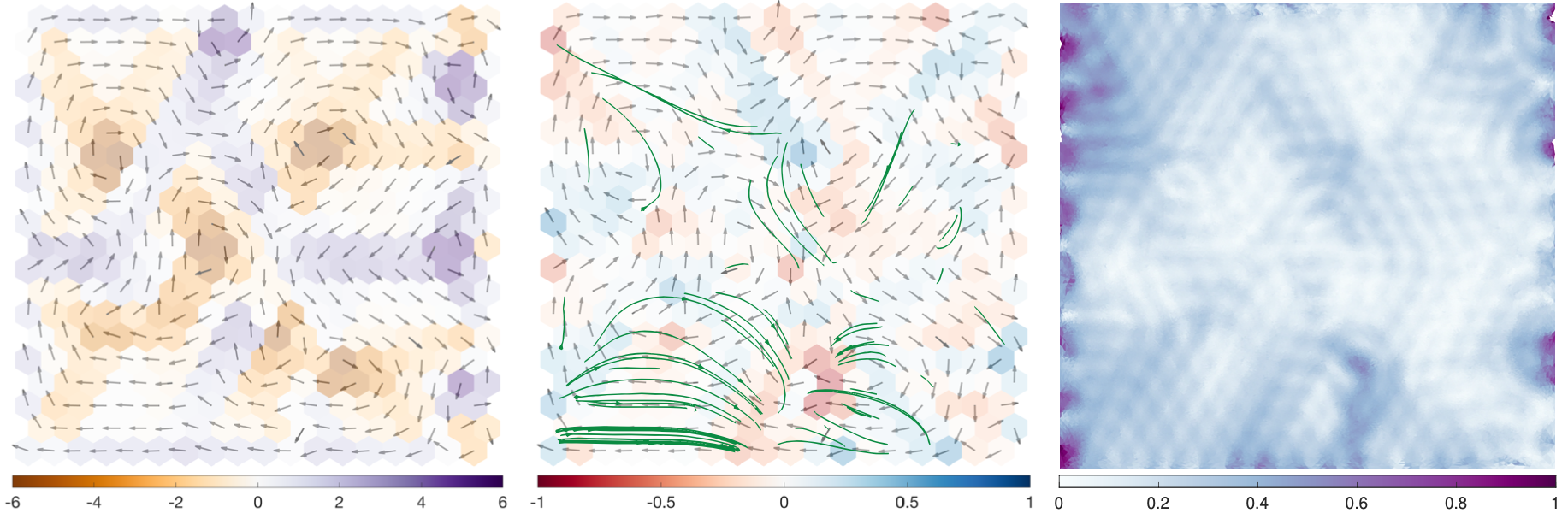


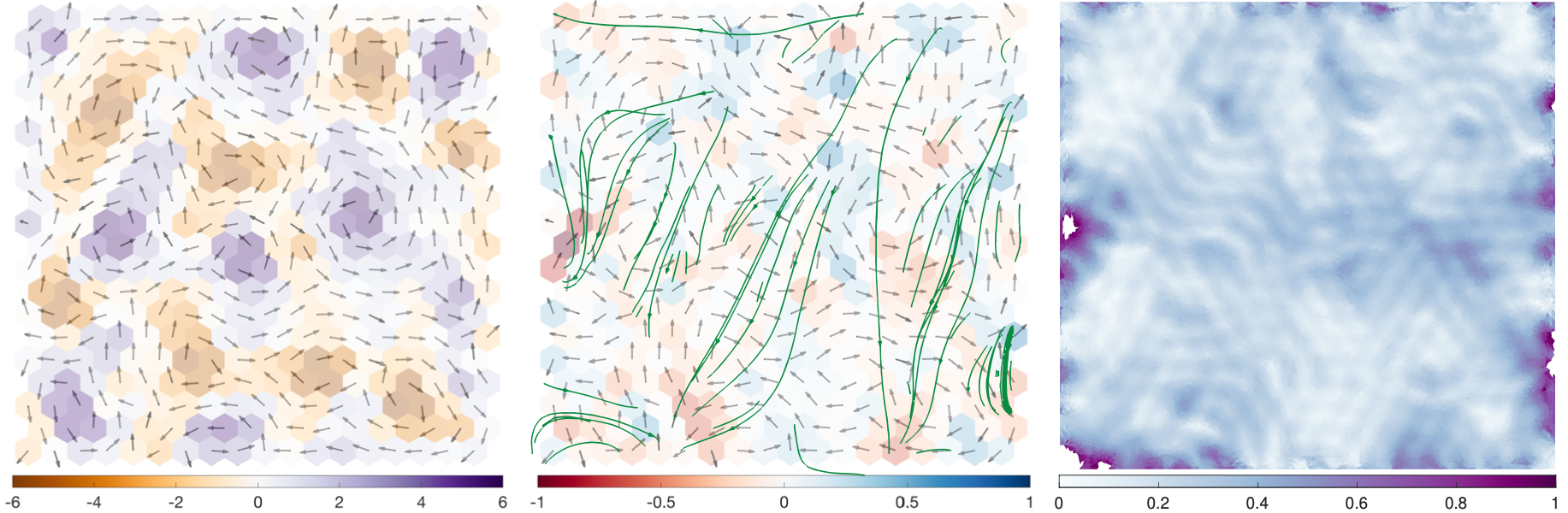


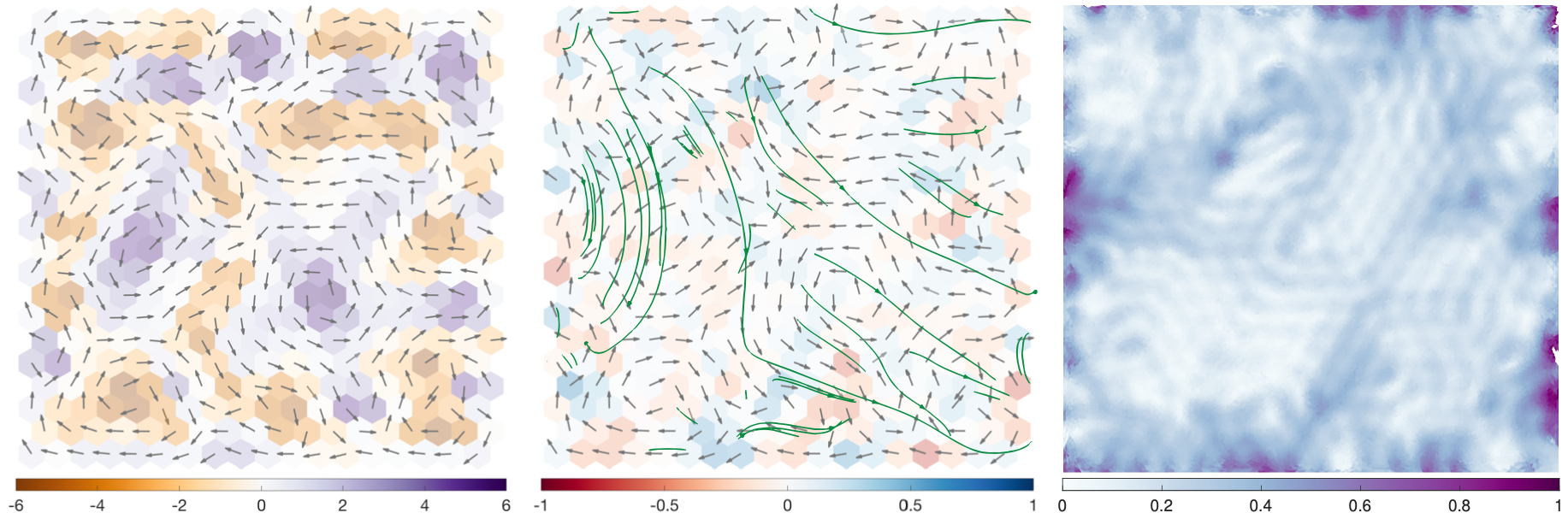


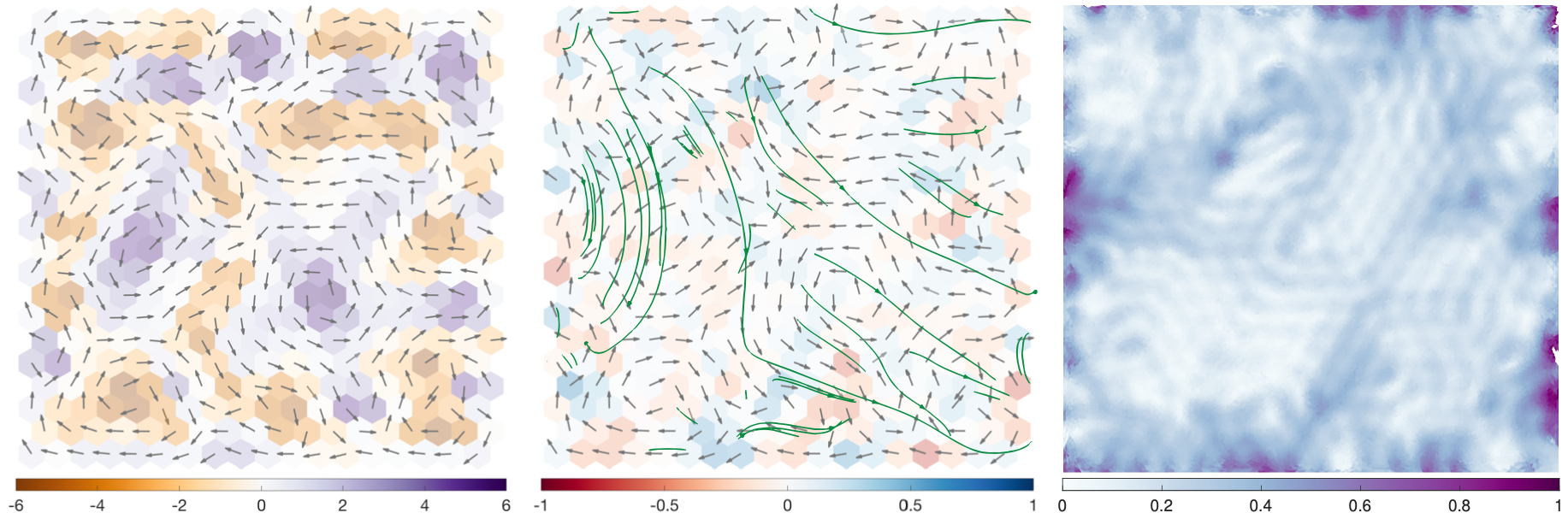


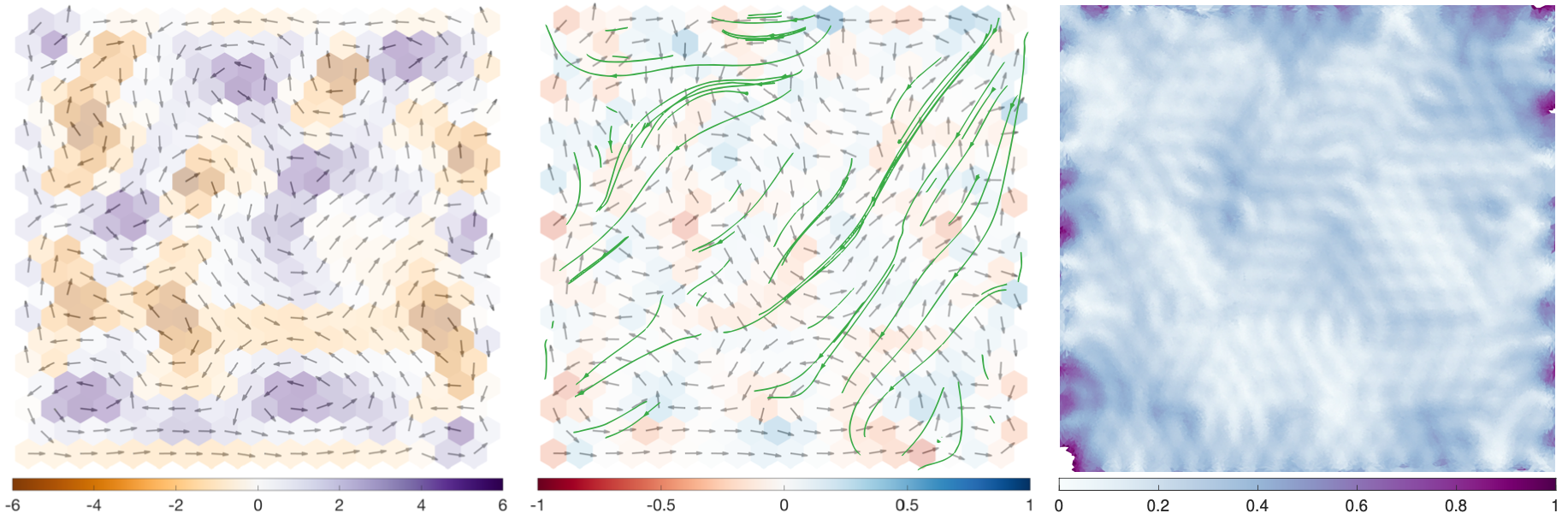


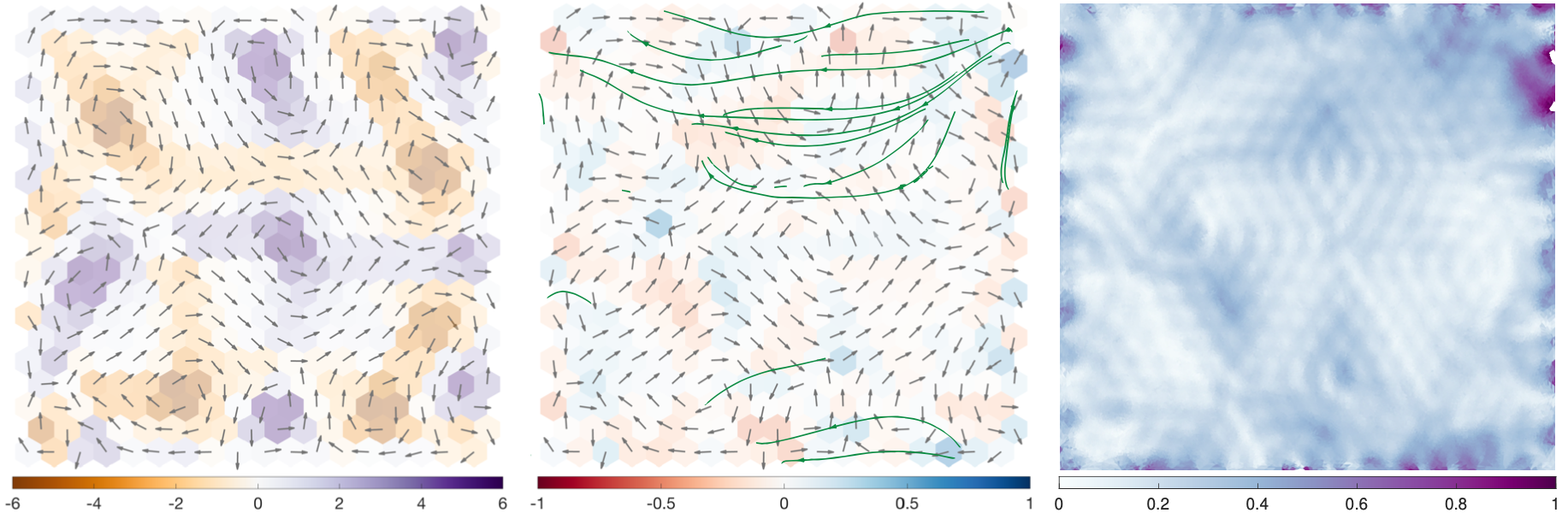


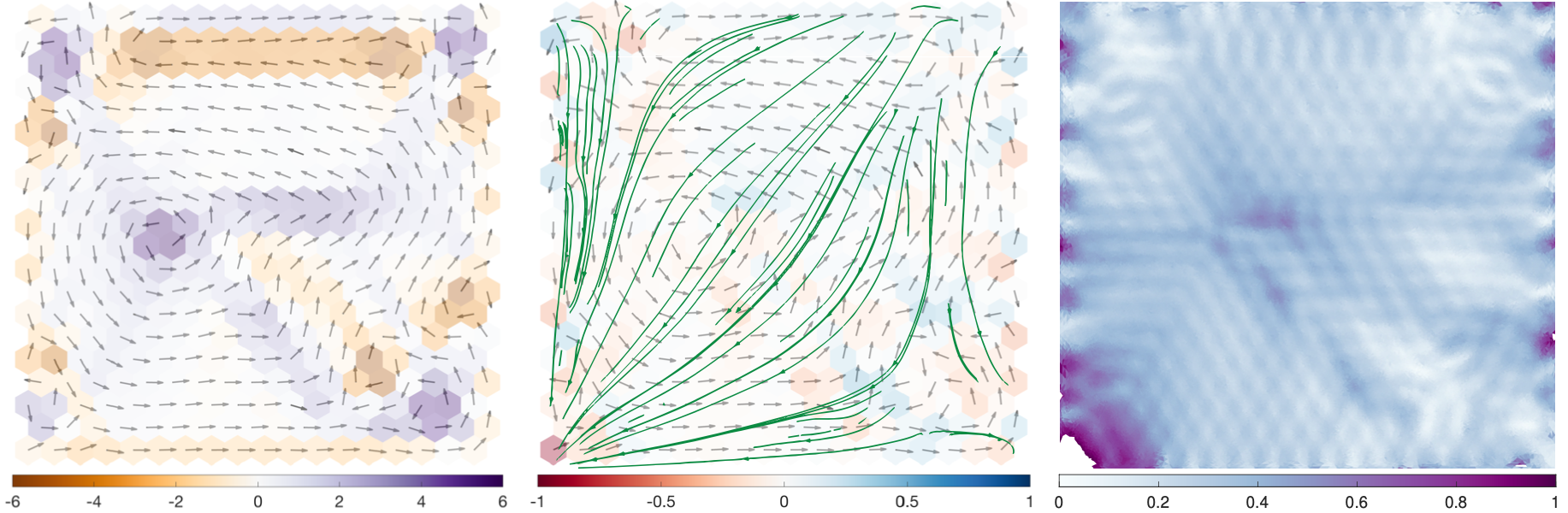


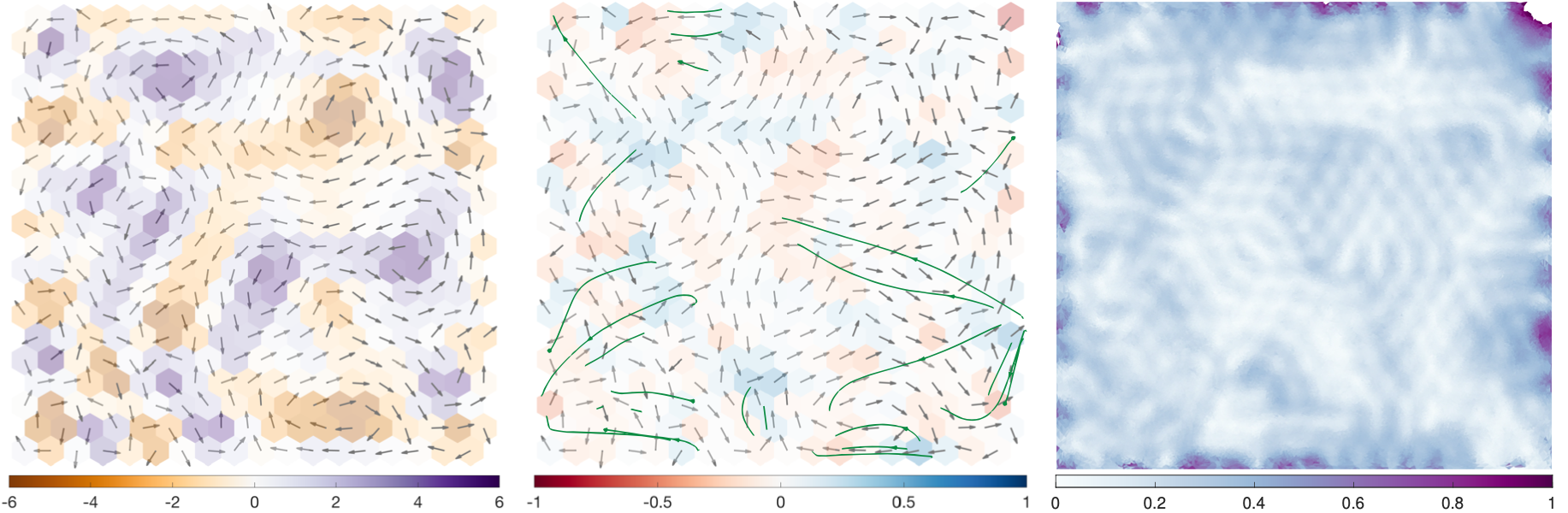


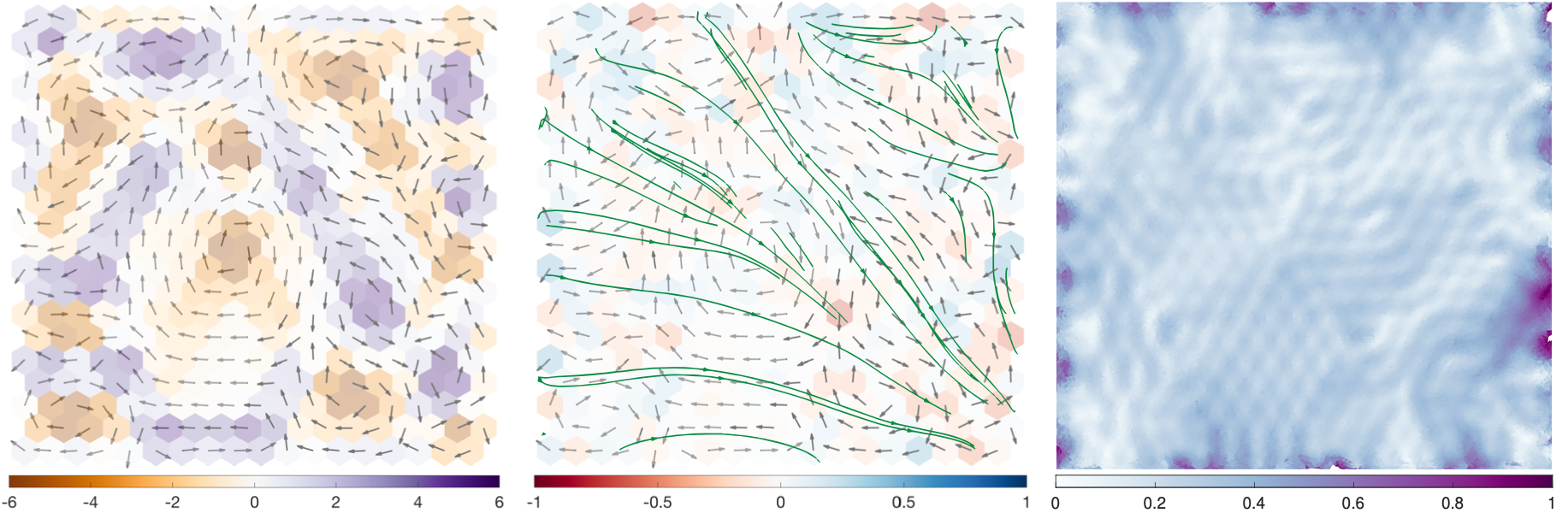


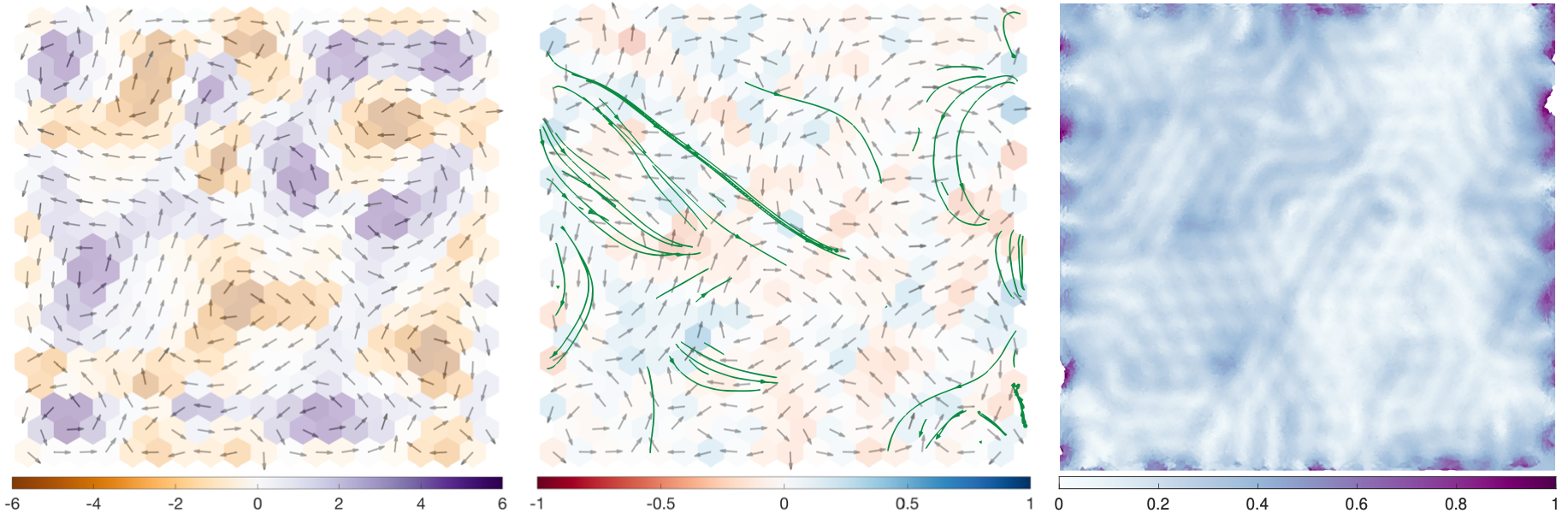


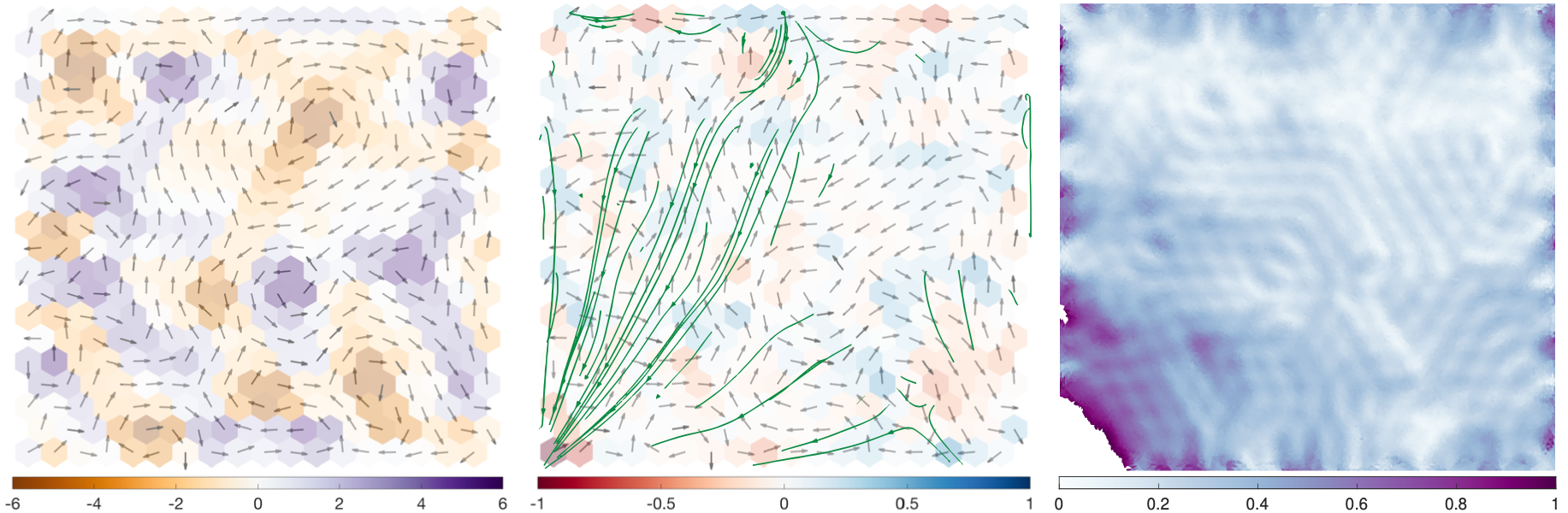


**Figure S3:** Every row shows different simulated spin glass states. Left: the in-plane magnetisation vector field, $M_{xy}$ (black arrows), and its curl, ${\nabla_{d}\times M}_{xy}$ (brown-purple colour scale). Middle: the in-plane magnetisation vector field, $M_{xy}$ (black arrows), and its divergence, ${\nabla_{d}\cdot M}_{xy}$ (red-blue colour scale). Green lines indicate the magnetic field lines where the magnetic flux is highest above the material plane (where $z>c$ with $c=2r$ being the cell pitch). Right: surface plot of the normalized magnetic field strength, $\vec{B}_{sg}$, at the material top surface $z=c$ (white-purple color scale). Regions with large in-plane divergence are sources (magnetic poles) of external magnetic field lines and coincide with regions of high magnetic field strength at the material surface.

Supplementary movie S4 shows local magnetisation of the sample by heating a sample under a non-uniform magnetic permanent field to align their magnetic particles in matching patterns.

Supplementary movie S5 shows a latching example with two samples that are magnetised with non-uniform magnetic permanent field. Samples latch onto each other at 90-degree rotation intervals.
